# Susceptibility of *Staphylococcus aureus* Clinical Isolates to Propolis Extract Alone or in Combination with Antimicrobial Drugs

**DOI:** 10.3390/molecules18089623

**Published:** 2013-08-12

**Authors:** Robert D. Wojtyczka, Arkadiusz Dziedzic, Danuta Idzik, Małgorzata Kępa, Robert Kubina, Agata Kabała-Dzik, Joanna Smoleń-Dzirba, Jerzy Stojko, Mieczysław Sajewicz, Tomasz J. Wąsik

**Affiliations:** 1 Department and Institute of Microbiology and Virology, School of Pharmacy and Division of Laboratory Medicine in Sosnowiec, Medical University of Silesia, ul. Jagiellońska 4, 41-200 Sosnowiec, Katowice, Poland; E-Mails: rwojtyczka@sum.edu.pl (R.D.W.); didzik@sum.edu.pl (D.I.); mkepa@sum.edu.pl (M.K.); jsmolen@sum.edu.pl (J.S.-D.); 2 Department of Conservative Dentistry with Endodontics, Medical University of Silesia, Pl. Akademicki 17, 41-902 Bytom, Katowice, Poland; E-Mail: adziedzic@sum.edu.pl; 3 Department and Institute of Pathology, Medical University of Silesia, ul. Ostrogórska 30, 41-200 Sosnowiec, Katowice, Poland; E-Mails: rkubina@sum.edu.pl (R.K.); adzik@sum.edu.pl (A.K.-D.); 4 Department Bioanalysis and Environmental Studies, School of Pharmacy and Division of Laboratory Medicine in Sosnowiec, Medical University of Silesia, ul. Kasztanowa 3A, 41-200 Sosnowiec, Katowice, Poland; E-Mail: jstojko@sum.edu.pl; 5 Institute of Chemistry, University of Silesia, ul. Szkolna 9, 40-006 Katowice, Poland; E-Mail: mieczyslaw.sajewicz@us.edu.pl

**Keywords:** methicillin-resistant *S. aureus* (MRSA), methicillin-sensitive *S. aureus* (MSSA), ethanolic extract of Polish propolis (EEPP)

## Abstract

The objective of this study was to assess *in vitro* the antimicrobial activity of ethanolic extract of Polish propolis (EEPP) against methicillin-sensitive *Staphylococcus aureus* (MSSA) and methicillin-resistant *Staphylococcus aureus* (MRSA) clinical isolates. The combined effect of EEPP and 10 selected antistaphylococcal drugs on *S. aureus* clinical cultures was also investigated. EEPP composition was analyzed by a High Performance Liquid Chromatography (HPLC) method. The flavonoid compounds identified in Polish Propolis included flavones, flavonones, flavonolols, flavonols and phenolic acids. EEPP displayed varying effectiveness against twelve *S. aureus* strains, with minimal inhibitory concentration (MIC) within the range from 0.39 to 0.78 mg/mL, determined by broth microdilution method. The average MIC was 0.54 ± 0.22 mg/mL, while calculated MIC_50_ and MIC_90_ were 0.39 mg/mL and 0.78 mg/mL, respectively. The minimum bactericidal concentration (MBC) of the EEPP ranged from 0.78 to 3.13 mg/mL. The *in vitro* combined effect of EEPP and 10 antibacterial drugs was investigated using disk diffusion method-based assay. Addition of EEPP to cefoxitin (FOX), clindamycin (DA), tetracycline (TE), tobramycin (TOB), linezolid (LIN), trimethoprim+sulfamethoxazole (SXT), penicillin (P), erythromycin (E) regimen, yielded stronger, cumulative antimicrobial effect, against all tested *S. aureus* strains than EEPP and chemotherapeutics alone. In the case of ciprofloxacin (CIP) and chloramphenicol (C) no synergism with EEPP was observed.

## 1. Introduction

The coagulase-positive *S. aureus* is a major pathogen responsible for various community-onset and hospital acquired infections. It causes skin and soft tissues infections, surgical site infections, bone infections pneumonia, bacteremia, endocarditis and joints infections [1,2,3]. The nasal carriage of *S. aureus* in healthy adults was reported to be around 20%–30% of the population. Colonization clearly increases the risk for subsequent infection [4,5]. At present the β-lactam antibiotics are the preferred drugs against *S. aureus* infections. Antibiotic resistant staphylococci strains are a major public health concern since the bacteria can easily circulate in the environment. *S. aureus* has developed resistance to the β-lactam antibiotics due to synthesis of chromosomal or plasmid encoded β-lactamases [4,5,6]. In comparison with methicillin-sensitive *Staphylococcus aureus* (MSSA), methicillin-resistant *Staphylococcus aureus* (MRSA) strains pose more problems, since invasive MRSA infections are associated with greater costs and limited treatment options [7].

Propolis (bee glue) is a natural resinous substance produced by honeybees (*Apis mellifera*) from plants’ buds and exudates, modified by addition of bees’ salivary secretions and wax [8,9]. It has been the subject of scientific interest for its diverse range of biological properties, including anti-inflammatory [10,11], immunomodulatory [12,13], anti-carcinogenic [14,15], antioxidant [16,17], radioprotective [18], antiviral [19], and antifungal [20] effects. What is more, the antibacterial activity of propolis has been confirmed in many studies [19,21,22,23,24,25]. The wide spectrum of biological activities of propolis has been attributed to its complex chemical composition, which is however, dependent on the plant species from which it is harvested, and may be influenced by the geographical and climatic factors, as well as the type of foraging honeybee [8,24,25,26,27]. In addition, it has been demonstrated that there were some differences in the antibacterial activity of propolis extracts depending on the collection region [26] and the races of honeybee [21]. Nevertheless, due to the complex and multidirectional mechanism of action of all types of propolis on bacterial cell [28,29], development of resistance to this substance is complicated and unlikely. For this reason, in the light of a rapid and widespread emergence of bacterial strains resistant to classic chemotherapeutics [30], beneficial, antimicrobial properties of propolis may be helpful in treatment of current bacterial infections. Studies on chemical composition of propolis from different geographical regions showed characteristic classes of compounds which correlated with selected plant sources. The most frequent techniques currently used for chemical composition analysis of propolis are spectrophotometric methods (UV-VIS, HP-TLC), high performance liquid chromatography (HPLC-DAD) coupled with different detectors, and gas chromatography-mass spectrometry (GC-MS) [31,32,33].

Many reports showed that *S. aureus* appears to be naturally susceptible to propolis [34]. Recent studies [8] revealed that propolis exerts synergistic effects with antibiotics, acting on the bacterial wall structure and ribosomes function, but it does not seem to interact with antibiotics acting on DNA or folic acid biosynthesis [35]. The mechanism of propolis antibacterial activity seems to be linked to some of its components. The potent bacteriostatic and bactericidal effects of propolis can be associated with their combined action manifested by inhibition of protein synthesis and bacterial growth by preventing cell division [36,37].

Particularly, the notion of using a combination of propolis and antibiotics to augment antibacterial therapy appears promising [34,38,39,40,41,42,43]. Some *in vitro* studies indicated evident synergism between propolis and antibiotics with a clear decrease of minimal inhibitory concentrations (MICs) for tested drugs [28,39], while in others this effect was less obvious [42], or not observed [41]. The results of the research carried out by Fernandes Junior *et al.*, demonstrated the synergism between EEP and those antibacterial agents that interfere with bacterial protein synthesis, e.g. chloramphenicol, gentamicin, netilmicin, tetracycline and clindamycin [38]. The antibacterial activity of propolis, together with its potential to enhance the antimicrobials efficacy due to possible synergistic interactions, could be especially advantageous in the treatment of *S. aureus* infections, which currently represent a significant burden on healthcare systems across the world [44].

Thus, since there is a great and still growing need for the enhancement of therapy of infections caused by different *S. aureus* strains, the objective of this study was to assess the in vitro antimicrobial activity of ethanolic extract of Polish propolis (EEPP) against MSSA and MRSA clinical isolates. To make sure that bacterial strains were identified unambiguously the Polymerase Chain Reaction-Restriction Fragment Length Polymorphism (PCR-RFLP) technique with XapI and Bsp143I restriction enzymes was applied. The investigation of the activity of the EEPP alone and in combination with selected antistaphylococcal drugs on MSSA and MRSA cultures was also evaluated.

## 2. Results and Discussion

**Figure 1:**
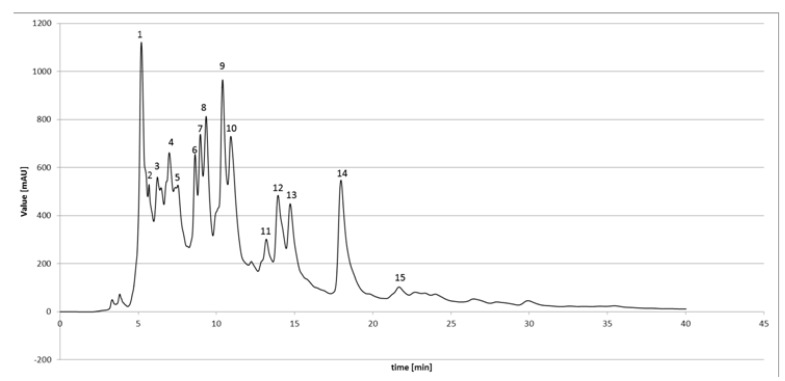
HPLC chromatogram of propolis ethanolic extract from Poland. 1-Cinnamic acid (Rt-5.20); 2-*p*-Coumaric acid (Rt-5.69); 3-Ferulic acid (Rt-6.23); 4-Gallic acid (Rt-6.98); 5-Caffeic acid (Rt-7.36); 6-Caffeic acid phenethyl ester (Rt-8.63); 7-Pinobaksin (Rt-8.97); 8-Kaempferol (Rt-9.34); 9-Apigenin (Rt-10.40); 10-Pinocembrin (Rt-10.92); 11-Quercetin (Rt-13.19); 12-Chrysin (Rt-13.93); 13-Galangin (Rt-14.72); 14-Acecetin (Rt-17.46); 15-Kampferide (Rt-21.67); Rt- retention time (min).

Quantitative analyses of ethanolic extracts of propolis are presented in Figure 1. Figure 1 represents a typical HPLC chromatogram of EEPP. Qualitative and quantitative analysis of selected flavonoids and phenolic acids was carried out, with the identification of pinocembrin, kaempferol, galangin, chrysin, apigenin, quercetin, gallic acid, ferullic acid, caffeic acid, caffeic acid phenethyl ester, *p*-coumaric acid and cinnamic acid. The flavonoid compounds identified in this study included flavones, flavanones, flavanolols, flavonols and chalcons. Most flavonoid compounds displayed the typical pattern of “poplar” propolis. Several studies showed that flavonoids have antimicrobial, anticancer, antioxidant action. It is known that the amount of phenolic and flavonoid constituents varies widely according to propolis types and seasonal factors. The HPLC analysis showed that flavonoid compounds of Polish propolis are similar to other ethanolic extracts of propolis, especially of European origin [17,24,26,28,36]. We made the conclusion that Polish propolis possesses significant amounts of biologically active compounds belonging to the classes of phenolic acids, and different classes of flavonoids (flavones/flavonols, flavanones/dihydroflavonols or other phenols) and can be subjected to other 'validated' methods for European poplar type propolis. Therefore, the multi-directional interactions among the various chemical compounds in propolis seem to be the essential biological activities when considering its antibacterial effects against pathogens.

**Figure 2:**
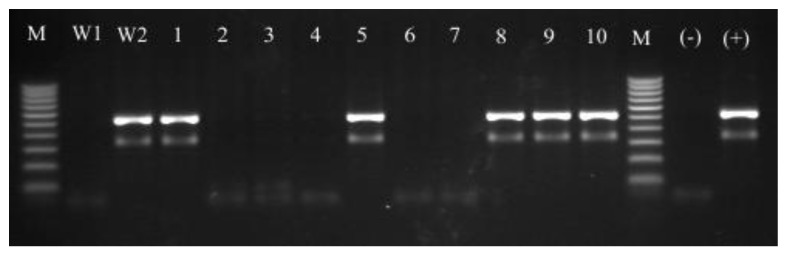
Identification of *mecA* gene fragment (533 bp). W1—*S.* *aureus* MSSA ATCC 25923; W2—*S. aureus* MRSA ATCC 43300; 1–10 *S.* *aureus* clinical isolates; M-100–1000 bp marker, (+) positive control, (−) negative control.

According to the standard and molecular method 10 clinical isolates were classified as belonging to the *Staphylococcus* *aureus* species (Table 1, Figure 2). 

Catalase positive, coagulase positive and mannitol fermenting staphylococcal strains were identified biochemically by the API STAPH system. The molecular species identification of coagulase-positive staphylococci by PCR-RFLP technique with *XapI* and *Bsp143I* restriction enzymes confirmed that all isolates were *S. aureus* strains.

**Table 1:** The phenotypic (Cefoxitin test) and genetic (PCR *mecA* gene) assessment of *S. aureus* strains susceptibility to methicillin.

Strain	Cefoxitin test	*mecA*	MSSA/MRSA
*S. aureus* 1	17 mm	+	MRSA
*S. aureus* 2	34 mm	−	MSSA
*S. aureus* 3	34 mm	−	MSSA
*S. aureus* 4	33 mm	−	MSSA
*S. aureus* 5	22 mm	+	MRSA
*S. aureus* 6	34 mm	−	MSSA
*S. aureus* 7	34 mm	−	MSSA
*S. aureus* 8	13 mm	+	MRSA
*S. aureus* 9	16 mm	+	MRSA
*S. aureus* 10	14 mm	+	MRSA
*S. aureus* W^1^	35 mm	−	MSSA
*S. aureus* W^2^	21 mm	+	MRSA

W^1^: *S. aureus* MSSA ATCC 25923; W^2^: *S. aureus* MRSA ATCC 43300.

The broth microdilution method was used to determine the MICs of the EEPP against 12 *S. aureus* strains. EEPP displayed varying degree of activity against *S. aureus* with MIC values ranging from 0.39 mg/mL to 0.78 mg/mL (Table 2, Table 3). The average MIC was 0.54 ± 0.22 mg/mL, MIC_50_ and MIC_90_ values were 0.39 mg/mL and 0.78 mg/mL, respectively. The MBC values of the EEPP ranged from 0.78 mg/mL to 3.13 mg/mL.

**Table 2:** Susceptibility of *S. aureus* MSSA strains to EEPP (MIC and MBC values in mg/mL).

Strain	2	3	4	6	7	W^1^
MIC EEPP (mg/mL)	0.39	0.39	0.78	0.78	0.78	0.39
MBC EEPP (mg/mL)	1.56	1.56	3.13	3.13	1.56	3.13

W^1^: *S. aureus* ATCC 25923.

**Table 3:** Susceptibility of *S. aureus* MRSA strains to EEPP (MIC and MBC values in mg/mL).

Strain	1	5	8	9	10	W^2^
MIC EEPP (mg/mL)	0.39	0.39	0.39	0.39	0.78	0.78
MBC EEPP (mg/mL)	3.13	3.13	0.78	0.78	3.13	3.13

W^2^: *S. aureus* MRSA ATCC 43300.

Values of MIC and MBC EEPP against MSSA strains were successively 0.59 ± 0.21 mg/mL and 2.5 ± 0.85 mg/mL. In the case of MRSA strains the MIC and MBC were as follows: 0.52 ± 0.2 mg/mL and 2.35 ± 1.21 mg/mL.

**Figure 3:**
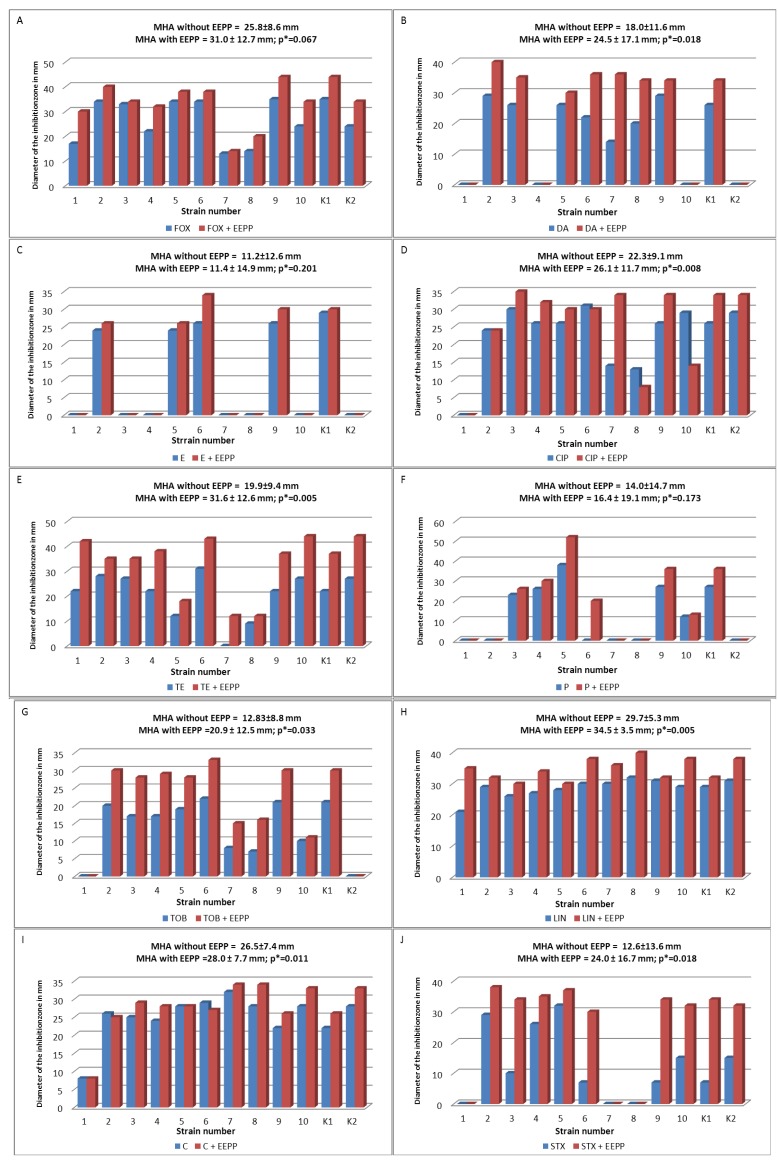
Inhibitory effect of 10 antimicrobial agents alone, and in combination with EEPP on 12 *Staphylococcus* strains evaluated by disk diffusion method. (**A**) FOX and FOX+EEPP; (**B**) DA and DA+EEPP; (**C**) E and E+EEPP; (**D**) CIP and CIP+EEPP; (**E**) TE and TE+EEPP; (**F**) P and P+EEPP; (**G**) TOB and TOB+EEPP; (**H**) LIN and LIN+EEPP; (**I**) C and C+EEPP; (**J**) STX and STX+EEPP. MHA: Mueller-Hinton Agar; MHA with EEPP: MHA plus one-fourth of MIC90 of EEPP; blue bars: diameters of the growth inhibition zones (in mm) for antibiotic alone; red bars: diameters of the growth inhibition zones (in mm) for combined effect of antibiotics and EEPP; FOX: Cefoxitin; DA: Clindamycin; E: Erythromycin; CIP: Ciprofloxacin; TE: Tetracycline; P: Penicillin; TOB: Tobramycin; LIN: Linezolid; C: Chloramphenicol; STX: Trimethoprim+Sulfamethoxazole; K1: *S. aureus* ATCC 25923; K2: *S. aureus* MRSA ATCC 43300; * Wilcoxon Signed-Rank Test, statistical significant level at *p* < 0.05.

The *in vitro* combined effect of EEPP and 10 antibacterial drugs (FOX, DA, E, CIP, TE, P, TOB, LIN, C, SXT) was tested using disk diffusion method-based analysis, and the results are shown in Figure 3. This analysis revealed synergism between EEPP and FOX, DA, TE, TOB, LIN, P, E, and SXT for all tested MSSA and MRSA strains. In the case of CIP and C synergism with EEPP was not observed.

**Figure 4:**
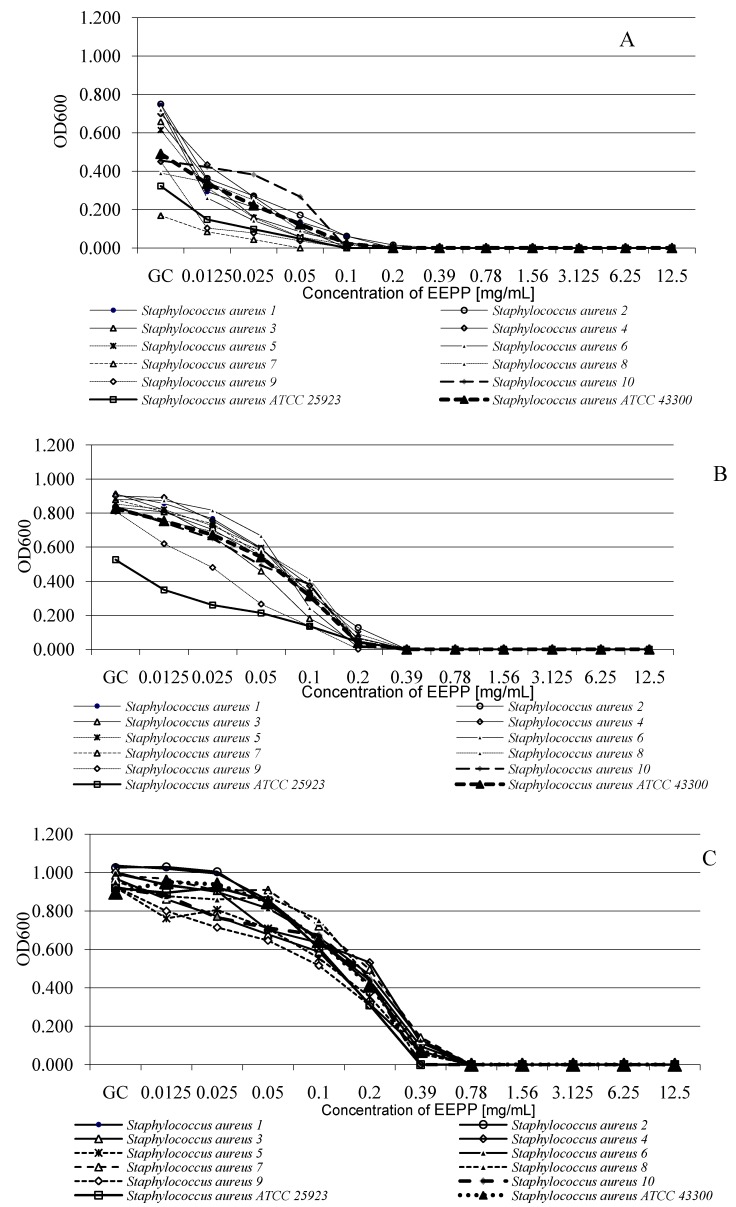
Growth of *S. aureus* strains in the presence of different EEPP concentrations. (**A**) After 6 h of incubation; (**B**) After 12 h of incubation; (**C**) After 24 h of incubation; GC: Growth control.

The analysis of bacterial growth after first 6 h of incubation showed that the growth of all strains in the medium supplemented with EEPP at concentrations ranging from 0.0125 to 0.39 mg/mL was not inhibited (Figure 4A). After 12 h of incubation, the growth of all strains was observed in the wells with the same range of EEPP concentrations (Figure 4B). After 24 h of incubation the growth of all MRSA and MSSA strains was observed at concentrations ranging from 0.0125 to 0.78 mg/mL (Figure 4C).

The three-way ANOVA indicated that the growth of all *S. aureus* strains was significantly affected by EEPP concentration (*p* < 0.001) and incubation time (*p* < 0.001). The interaction between these factors was also significant (*p* < 0.001). The EEPP concentration effect (40.12%), incubation time (21.10%), and interaction between concentration and incubation time (20.23%) explained most of variance (Table 4).

**Table 4:** Multivariate analysis of variance by three-way ANOVA of *S. aureus* strains susceptibility to EEPP

Source of variation	df	Sum of squares	Mean squares	Variance explained (%)	F	*p*
Strain (S)	11	0.67	0.06	0.71	27.2	<0.001
Time (T)	2	19.8	9.9	21.10	4414.6	<0.001
Concentration (C)	11	37.65	3.42	40.12	1526.5	<0.001
S x T	22	1.46	0.07	1.56	29.5	<0.001
S x C	121	6.74	0.06	7.18	24.8	<0.001
T x C	22	18.99	0.86	20.23	384.9	<0.001
SxTxC	242	8.54	0.04	9.01	15.7	<0.001

In this study; MIC EEPP values ranged from 0.39 to 0.78 mg/mL, while MBC values ranged from 0.78 to 3.13 mg/mL. For MRSA strains, average MIC values were 0.52 ± 0.2 mg/mL, and for MSSA strains 0.59 ± 0.21 mg/mL. The MBC average values were similar for MRSA and MSSA strains, and found to be 2.35 ± 1.21 mg/mL and 2.35 ± 0.86 mg/mL, respectively. The MIC_AB_ average values were similar for MRSA and MSSA strains, and found to be 0.41 ± 0.22 mg/mL and 0.42 ± 0.31 mg/mL, respectively. The ethanol in EEPP was not responsible for the anti-staphylococcal activity of EEPP when compared to 70% ethanol control.

It was previously reported that ethanolic extract of propolis showed various inhibitory activities against different microorganisms when tested *in vitro* [22,26,34,43,45,46] and *in vivo* [47]. In the study of Takaisi-Kikuni and Schilcher [29], some mechanisms of propolis activity on bacterial growth have been discussed. Havsteem [36] and Oksuz *et al.* [37] suggested that the specific propolis ingredients inhibit protein synthesis and bacterial growth by preventing cell division, resulting in the formation of pseudo-multicellular bacterial forms. Galangin and caffeic acid from EEP are enzymatic inhibition agents responsible for an inhibition of bacterial growth and proliferation. In addition, some active substances composing propolis may disorganize the cytoplasmic membrane and cell wall, with the effect of a partial bacteriolysis. Flavonoids affect bacterial membrane potential and cause permeability alteration within the inner microorganisms membrane [28].

Numerous studies have shown that the antimicrobial effect of EEP may vary according to the geographic region, as the propolis samples from different locations may exhibit different chemical composition. Seidel *et al.* [26] presented an evaluation of the antibacterial activity of 40 propolis samples collected from various locations worldwide. The propolis samples from Africa and Asia showed moderate activity, with MICs ranging from 0.0156 to >0.5 mg/mL and 0.0078 to >0.5 mg/mL, respectively. Samples from North and South America and samples collected in Europe displayed similar anti-staphylococcal activity, with the MIC values in the range of 0.125 to >0.5 mg/mL. Kilic *et al.* analyzing EEP effect on MRSA strains reported that MIC values of propolis samples from three different regions of Turkey, *i.e.* Mamak and two Kemaliye locations varied and amounted to 0.018 ± 0.008, 0.162 ± 0.073, and 0.101 ± 0.040 mg/mL, respectively [48].

The results presented by Berretta *et al.* [49] demonstrated that the MBC of three samples of standardized propolis extract tested against *S. aureus* ATCC 25923 and *S. aureus* ATCC 43300 were in the range from 6.96 to 7.02 mg/mL and 3.48 to 3.51 mg/mL respectively.

In the present study for the same reference strains the EEPP MBC values were lower (3.13 mg/mL) showing higher bactericidal activity of Polish propolis’ extract. The diverse antimicrobial activity of EEP shown by many authors and in our manuscript can be due to the differences in the origin of propolis, and its qualitative and quantitative composition.

Wojtyczka *et al.* [25] showed the antistaphylococcal activity of the Polish EEPP against 11 *S. epidermidis* strains using the broth microdilution method. In this study EEPP displayed varying degrees of activity against CoNS with MIC in the range of 1.56–0.78 mg/mL. The average MIC was 1.13 ± 0.39 mg/mL while calculated MIC_50_ and MIC_90_ were 0.78 mg/mL and 1.56 mg/mL, respectively.

Investigated hospital-acquired clinical isolates revealed varying susceptibility to antibiotics. Apart from the common resistance to β-lactam antibiotics, MRSA may often demonstrate an increased resistance towards other groups of antibiotics. Our study on the influence of EEPP on MSSA and MRSA strains showed no significant differences, both MIC and MBC values obtained for these strains were relatively similar. This may lead to the conclusion that EEPP demonstrates an essential anti-staphylococcal activity not associated with beta-lactam antibiotics. Gonsales et al. observed the antibacterial activity of propolis using a diffusion method, in which EEP inhibited *S. aureus* growth, with diameters of the growth inhibition zones ranging from 8 to 13 mm [50]. Similar results were obtained by Stepanovic *et al*. In their study, the zone of microbial growth inhibition for 13 propolis samples obtained from different geographic regions of Serbia were in the range from 9 to 13 mm [43].

To estimate the combined effect of EEPP with a set of widely used antibiotics, the propolis in concentrations which did not by itself inhibit bacterial growth were used on solid MHA plates in the disk diffusion assay. 

MIC is usually measured by the broth dilution method and the convenient disk diffusion method, however the convenient disk diffusion method-based analysis is also used frequently to estimate drug susceptibility. These methods, using visual evaluation of bacterial growth in the presence of a tested compound, do not take into the consideration the cell metabolism. Therefore, a special technique is required for the assessment of bactericidal activity.

In our study, the addition of EEPP at a concentration equal to ¼ of MIC_90_ (0.2 mg/mL) to MHA medium significantly increased sensitivity of *S. aureus* strains to DA (*p* = 0.018), TET (*p* = 0.005), TOB (*p* = 0.033), LIN (*p* = 0.005), and SXT (*p* = 0.018). Such synergy between EEPP and antibiotics was also observed for FOX (*p* = 0.067), E (*p* = 0.201) and P (*p* = 0.173), but the values did not reach statistical significance. In particular, a strong combined effect was observed in the case of TET and SXT, for which diameters of the growth inhibition zones on MHA medium with addition of EEPP were larger by 11–12 mm that the measured on MHA medium without EEPP. For the remaining antibiotics (FOX, DA, CIP, TOB, LIN) tested in combination with EEPP, the diameters of the growth inhibition zones were larger by 4–8 mm than that measured on MHA medium without EEPP.

Synergistic effects of propolis and antibiotics on the growth of *S. aureus* have been reported previously by Krol *et al.* [34]. However, this effect was significant only at a propolis concentration of 0.6 mg/mL, while in our experiment the effect appeared at 0.2 mg/mL EEPP concentration.

EEP is known to contain a number of antimicrobial compounds, such as polyphenols and flavonoids. The antimicrobial and resistance modifying potentials of natural compounds have been reported by Cushnie and Lamb [51]. This suggests that the synergy with antibiotics observed in this study could be attributed to such compounds. Some of these compounds, like polyphenols, have been shown to exert their antibacterial action through membrane perturbations. This perturbation of the cell membrane coupled with the action of β-lactams on the transpeptidation of the cell membrane could lead to the enhanced antimicrobial effect [52].

## 3. Experimental

### 3.1. Ethanolic Extract of Polish Propolis (EEPP)

Propolis samples produced by honeybees (Apis mellifera) from an apiary in Kamianna near Nowy Sącz in southern Poland constituted the material for the study. This area is primarily rich in black poplar (*Populus nigra*), birch (*Betula alba*), alder (*Alnus glutinosa*), beech (*Fagus sylvatica*) and horsechestnut (*Aesculus hippocastanum*). Hand collected propolis were kept desiccated in the dark prior to its processing. Propolis was subjected to 14 days of extraction in order to obtain the ethanol extract of propolis, which was later dissolved in 70% ethanol to obtain a 100 mg/mL working concentration. Briefly, the samples were ground mechanically and bottled in 10 g portions. The 10 g portions were put into flask and 100 g of 70% ethanol (w/v) was added. The flask was placed on a rotary shaker in a dark, closed room for two weeks in room temperature. After this period, the extract was cooled in 4 °C for 24 h in order to precipitate all insoluble particles, which were removed from the propolis extract by filtration through filter paper (Whatman no. 4). Next, the obtained filtrate was evaporated to dryness at 40 °C using a rotary vacuum evaporator. In order to prepare a working concentration, the brown colored viscous substance was dissolved in 70% ethanol. 

### 3.2. HPLC-DAD Analysis of EEPP

To determine the chemical composition of EEPP a high performance liquid chromatography method was applied. Analysis was run on a Varian 920-LC HPLC (Harbor City, CA, USA), equipped with a 900-LC model autosampler, gradient pump, 330 model DAD, and the Galaxie software for data acquisition and processing. Separation was achieved using gradient mode on a Pursuit C18 (5 μm particle size) column (250 × 4.6 mm id; Varian; Cat. no. 1215–9307) using water, formic acid (95:5, v/v) (solvent A) and acetonitrile (solvent B). The elution was carried out at a flow rate of 0.6 mL/min. The separations were performed with a gradient elution: 20%–30% solvent B (for 15 min), 30% solvent B (15–28 min), 30%–80% solvent B (28–50 min), 80% solvent B (50–54 min), 80%–40% solvent B (54–60 min) and 40%–20% solvent B (60–65 min) The detection was monitored at 254 and 340 nm and the components identified by comparison with standards acquired commercially or isolated during previous work. EEPP were filtered with a 0.22 μm filter (Millipore) prior to injection of 20 μL into the HPLC system. Standard mixtures in ethanol containing chrysin, apigenin, acacetin, galangin, kaempferol, kaempferid, quercetin, pinostrombin, gallic acid, ferulic acid, cinnanic acid, *o*-coumaric acid, *m*-coumaric acid, *p*-coumaric acid, caffeic acid and caffeic acid phenylethyl ester (CAPE) were prepared from standard stock solutions (each at a concentration of 0.1 mg/mL in ethanol). All phenolic compounds were purchased from Carl Roth GmbH (Karlsruhe, Germany) and Sigma Chemical Company (St. Louis, MO, USA).

### 3.3. Bacterial Strains

The antibacterial activity of EEPP was assessed against ten coagulase-positive *S. aureus* strains isolated from blood clinical samples, and two reference strains of *S. aureus* ATCC 25923 and *S. aureus* ATCC 43300 as the MSSA and MRSA positive controls, respectively. Isolates were identified by conventional methods, including Gram staining, colony morphology, hemolysis, test for catalase, coagulase activity and anaerobic fermentation of mannitol. Catalase positive and coagulase positive staphylococcal isolates were identified by the API STAPH system (bioMerieux, Marcy l’Etoile, France) according to the manufacturer’s instructions. All bacterial strains were stored in Trypticase Soy Broth (TSB) medium with 20% of glycerol at −86 °C, until further analyses were performed.

### 3.4. Molecular Identification of Isolated Strains—PCR-RFLP Analysis of *dnaJ* Gene

For molecular analyses, bacterial genomic DNA was extracted with the GeneMATRIX Tissue & Bacterial DNA Purification KIT (EuRx Ltd., Gdańsk, Poland) according to the manufacturer’s recommendations and used for the PCR-RFLP analysis as described previously by Shah *et al.* [53]. Briefly, the *dnaJ* primers SA-(F) (5′-GCC AAA AGA GAC TAT TAT GA-3′) and SA-(R) (5′-ATT GYT TAC CYG TTT GTG TAC C-3′) were used to amplify the *dnaJ* gene fragment. The PCR amplification was performed using 10 × PCR RED master mix kit (BLIRT SA, Poland) in a MJ Mini Personal Thermal Cycler (Bio-Rad, Hercules, CA, USA). The PCR products were visualized under UV light after the electrophoretic separation in a 1.5% agarose gel (Promega, Madison, WI, USA) with ethidium bromide (EtBr). To identify isolated staphylococci strains, the PCR products were treated with 10 U of the *XapI* or *Bsp143I* restriction enzymes to obtain the species-specific restriction profiles. Digestions were performed in a total volume of 15 µL, with 5 µL of the PCR products, 1 µL of reaction buffer and 10 U of the *XapI* or *Bsp143I* endonucleases (Fermentas, Vilnius, Lithuania) for 3 h at 37 °C [54]. The obtained fragments were separated in a 2% agarose gel with EtBr (Promega), visualized under the UV light, and checked for size against 1 Kb HypeLadderIV (BLIRT SA, Gdańsk, Poland) molecular weight marker.

### 3.5. MSSA and MRSA Detection

#### 3.5.1. Cefoxitin Test

MRSA isolates were detected using cefoxitin disk diffusion method. A colony suspension equivalent to 0.5 McFarland was inoculated to Mueller-Hinton agar (MHA—BTL, Łódź, Poland), with a 30 μg cefoxitin disk (EMAPOL, Gdańsk, Poland) and interpreted after 20 h of incubation at 35 °C. MRSA strains were identified using a breakpoint of ≤21 mm zone diameter size for cefoxitin disks.

#### 3.5.2. The *mecA* Gene Detection

PCR detection of the *mecA* gene was performed with the primers and reaction conditions described previously by Murakami *et al.* [55]. *MecA* primers complementary to the penicillin binding protein (PBP2’) coding region (F) (5’-AAA ATC GAT GGT AAA GGT TGG C-3’) and (R) (5’-AGT TCT GCA GTA CCG GAT TTG C-3’) were used. The PCR amplification was performed using 10 × PCR RED master mix kit (BLIRT SA) in a MJ Mini Personal Thermal Cycler (Bio-Rad). The PCR products of 533 bp were detected under UV light after electrophoretic separation in a 1.5% agarose gel (Promega) with EtBr.

### 3.6. Antibacterial Susceptibility Testing

#### 3.6.1. Disk Diffusion Method

All isolates were tested for antimicrobial susceptibility by the disk diffusion method-based analysis, using MHA and commercially available disks containing an antimicrobial agent according to the EUCAST recommendations [56]. For disk diffusion testing, 90 mm plates with the agar medium were inoculated by swabbing the agar with a swab soaked in a bacterial suspension of 1 × 10^8^ cells/mL. Disks (EMAPOL) containing penicillin (P) 1 IU, erythromycin (E) 15 µg, clindamycin (DA) 2 µg, cefoxitin (FOX) 30 µg, ciprofloxacin (CIP) 5 µg, tobramycin (TOB) 10 µg, chloramphenicol (C) 30 µg, linezolid (LIN) 10 µg, tetracycline (TE) 30 µg or trimethoprim+sulfamethoxazole (SXT) 1.25 + 23.75 µg were used for the analysis of antimicrobial susceptibility.

The combined effect of antibiotics and EEPP was studied using plates with MHA plus one-fourth of MIC90 of EEPP, which was considered as a sub-inhibitory concentration [38,57]. Disks were placed onto agar surface and gently pressed to ensure contact using sterile forceps. Plates were incubated at 35 °C for 20 h in air. The susceptibility testing of each antibiotic for each isolate and the reference strains was performed in triplicates. After the incubation period diameters of the growth inhibition zones (in mm) were measured for each strain, and the mean values were calculated.

#### 3.6.2. Microdilution Method

MICs of EEPP were determined by the broth microdilution liquid and growth inhibition method. Growth inhibition assays were performed in the sterile Nunc 96-well plates, in a final volume of 200 μL [58,59]. The cell concentrations were estimated from the optical densities at 600 nm wavelength with the formula CFU/mL = *A*_600_ (3.8 × 10^8^), where CFU was the number of colony-forming units. One hundred microliters of mid-logarithmic-phase bacterial cultures (5 × 10^5^ CFU/mL) in Mueller-Hinton broth (MHB) was added to 100 μL of serially diluted EEPP (12.5 to 0.01 mg/mL). Wells containing MHB with bacterial inoculum only served as the bacterial growth control (GC). Additional controls included MHB alone (medium sterility control), MHB with different concentrations of EEPP, and MHB with different concentration of 70% ethanol and bacterial inoculum. All samples were prepared in triplicates. Microplates were incubated at 37 °C for 20 h, and the bacterial cell growth was assessed by measuring the optical density of cultures at 600 nm wavelength with a Multiskan EX microplate reader (Thermo Electron Corp., Vantaa, Finland) [60,61].

The MICs were defined as the lowest concentration that completely inhibits bacterial growth [58,59,61]. The MIC_50_ represents the MIC value at which ≥50% of the isolates in a test population are inhibited and is equivalent to the median MIC value. The MIC_90_ represents the MIC value at which ≥90% of the strains within a test population are inhibited; the 90^th^ percentile [62].

The MBCs were expressed as the lowest concentration of an antimicrobial agent (mg/L), that *in vitro* reduces the number of bacteria by 99.9%, within a defined period of time [56]. To determine the MBC value of EEPP 100 µL aliquots from each EEPP dilution, were transferred into MHA plates and incubated at 37 °C for 20 h. After incubation period, the number of colonies was calculated, and the initial CFU/well retrospectively determined [63].

### 3.7. Statistical Analyses

To determine the percentage of the variation attributable to the factors such as bacterial strains, time, and concentrations the results concerning the bacterial growth were analyzed by a three-way analysis of variance (ANOVA). The results from synergism assay were submitted to the Wilcoxon Signed-Rank Test comparing the values (mm) of the inhibitory zone in the disk diffusion method. All statistical analyses were performed using the Statistica 10.0 PL software package, assuming the statistical significance level of *p* < 0.05.

## 4. Conclusions

Our data showed that clinical strains of MSSA and MRSA with different drug resistance patterns were susceptible to EEPP. The action of EEPP was manifested by both growth inhibition of microorganisms (MIC) and bactericidal (MBC) activity. What is more, the observed synergistic effects of EEPP with commonly used antibiotics should induce further research on including EEPP in antimicrobial therapy schemes to augment their potential toward clinical strains of *S. aureus.*

Despite the fact, that the results presented in our study are promising, further randomized studies are needed to determine the clinical effectiveness of the synergistic action of propolis and antimicrobial drugs on staphylococci infections resistant to standard treatments. The propolis extract may facilitate and augment the antibiotic action by correcting the pharmacokinetic and pharmaco-dynamic properties and thus potentiate its biological action. 
